# Supplementary material for: Evaluation of a FlpA Glycoconjugate Vaccine with Ten N-Heptasaccharide Glycan Moieties to reduce Campylobacter jejuni Colonisation in Chickens
Source: Vaccines (Basel). 2024 Apr 9;12(4):395. doi: 10.3390/vaccines12040395 (PMC11054393; doi:10.3390/vaccines12040395)
Supplement: Supplementary file 1 [file vaccines-12-00395-s001.zip › vaccines-2893774-supplementary.pdf]

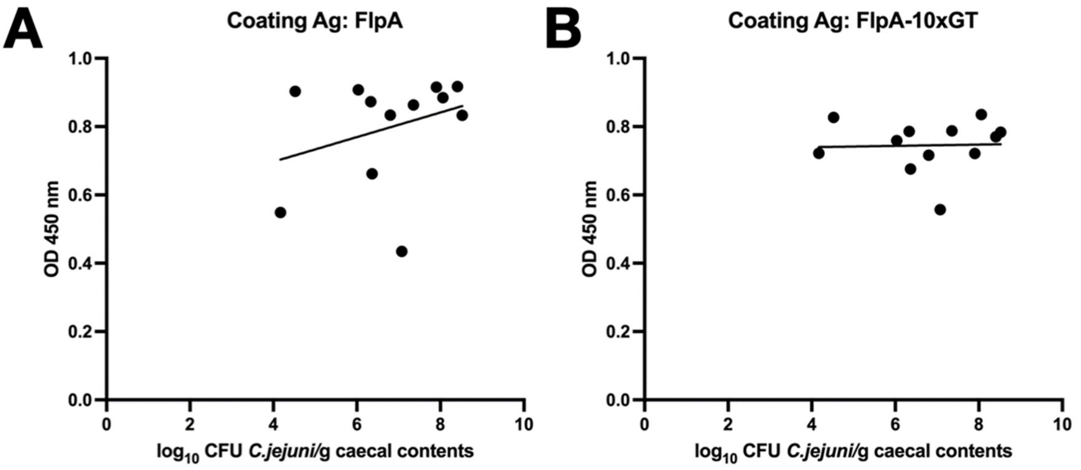

Supplementary Figure S1. Antigen-specific serum Ig levels and caecal *C. jejuni* load are not correlated in chickens vaccinated with FlpA or FlpA-10×GT.

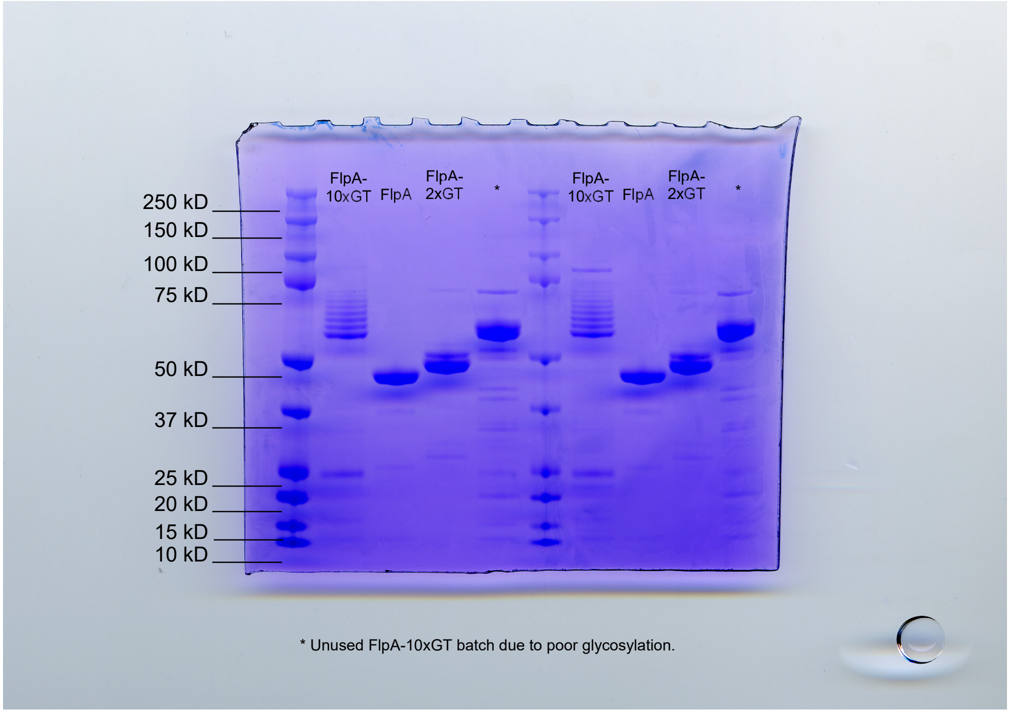

Supplementary Figure S2. Coomassie stained SDS-PAGE of subunit vaccines used for the study. Original scanned image of the gel shown in Figure 1A.

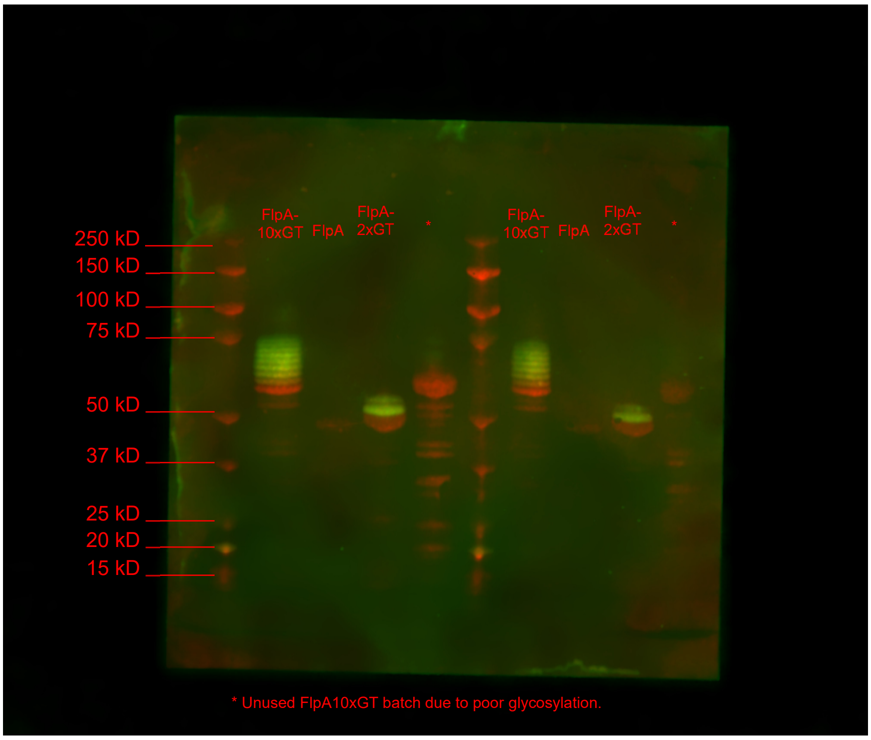

Supplementary Figure S3. Western blot stained with biotinylated soybean agglutinin (green) and anti-6×His antibody (red) to detect the glycan and protein portions respectively in the subunit vaccines used for the study. Original scanned image of the gel shown in Figure 1B.

Number of occupied glycosylation sites in band	MW (kD)	Average anti-6xHis fluorescence (FU)	Average lectin fluorescence (FU)
Unglycosylated	51	3.36 ± 1.82	0.00 ± 0.00
1	52	1.60 ± 0.68	1.95 ± 1.31
2	53	1.70 ± 0.99	4.03 ± 1.73
3	55	1.60 ± 0.96	4.75 ± 1.29
4	56	1.46 ± 0.75	4.83 ± 1.26
5	58	1.40 ± 0.35	4.69 ± 0.97
6	59	1.03 ± 0.29	3.66 ± 1.79
7	60	0.83 ± 0.45	2.71 ± 1.93
8	62	0.50 ± 0.28	1.70 ± 1.23
9	63	0.25 ± 0.21	0.63 ± 0.44
10	65	0.00 ± 0.00	0.00 ± 0.00

Supplementary Table S1. Densitometry measurements used to calculate the glycan dose. The level of glycosylation of the vaccine preparations and the *N*-glycan dose administered to the birds was estimated semi-quantitatively by densitometry. The fluorescence intensity of each band was measured in 3 repeats of identical western blots stained with a biotinylated lectin and a

fluorescent anti-6×His antibody. The number of occupied glycosylation sites was estimated from the predicted molecular weight (50.47 kDa for FlpA and 1.4 kDa per glycan moiety). The proportion of glycosylated protein was estimated to be 77%. Molecular weight is abbreviated to MW. Arbitrary fluorescence units are abbreviated to FU. The formula used to calculate the glycan dose per bird where n = number of occupied glycosylation sites was:

$$Glycan\ per\ dose\ (\mu g) = \sum_{n=1}^{10}\left(\frac{MW\ of\ glycan \times n}{MW\ of\ protein} \times \frac{Lectin\ fluorescence\ of\ the\ n^{th}\ band}{\sum(Lectin\ fluorescence\ of\ all\ bands)} \times \frac{\sum(Anti6xHis\ fluorescence\ of\ glycosylated\ bands)}{\sum(Anti6xHis\ fluorescence\ of\ all\ bands)} \times 100\mu g\ (dose)\right)$$
